# *Plicosepalus acacia* Extract and Its Major Constituents, Methyl Gallate and Quercetin, Potentiate Therapeutic Angiogenesis in Diabetic Hind Limb Ischemia: HPTLC Quantification and LC-MS/MS Metabolic Profiling

**DOI:** 10.3390/antiox10111701

**Published:** 2021-10-27

**Authors:** Asmaa R. Abdel-Hamed, Eman T. Mehanna, Reem M. Hazem, Jihan M. Badr, Dina M. Abo-Elmatty, Maged S. Abdel-Kader, Marwa S. Goda

**Affiliations:** 1 Department of Biochemistry, Faculty of Pharmacy, Suez Canal University, Ismailia 41522, Egypt; asmaa.ramdan@pharm.suez.edu.eg (A.R.A.-H.); eman.taha@pharm.suez.edu.eg (E.T.M.); dina_abouelmouti@pharm.suez.edu.eg (D.M.A.-E.); 2 Department of Pharmacology and Toxicology, Faculty of Pharmacy, Suez Canal University, Ismailia 41522, Egypt; reem_ahmed@pharm.suez.edu.eg; 3 Department of Pharmacognosy, Faculty of Pharmacy, Suez Canal University, Ismailia 41522, Egypt; gehan_ibrahim@pharm.suez.edu.eg (J.M.B.); marwa_saeed@pharm.suez.edu.eg (M.S.G.); 4 Department of Pharmacognosy, College of Pharmacy, Prince Sattam Bin Abdulaziz University 173, Al-Kharj 11942, Saudi Arabia; 5 Department of Pharmacognosy, College of Pharmacy, Alexandria University, Alexandria 21215, Egypt

**Keywords:** *Plicosepalus acacia*, LC-MS/MS, methyl gallate, quercetin, HPTLC, hind limb ischemia, therapeutic angiogenesis, VEGF, miR-146a

## Abstract

*Plicosepalus acacia* (Fam. Loranthaceae) has been reported to possess hypoglycemic, antioxidant, antimicrobial, and anti-inflammatory effects. Liquid chromatography combined with tandem mass spectrometry (LC-MS/MS) analysis revealed the presence of a high content of polyphenolic compounds that are attributed to the therapeutic effects of the crude extract. In addition, methyl gallate and quercetin were detected as major phytomedicinal agents at concentrations of 1.7% and 0.062 g%, respectively, using high-performance thin layer chromatography (HPTLC). The present study investigated the effect of the *P. acacia* extract and its isolated compounds, methyl gallate and quercetin, on hind limb ischemia induced in type 1 diabetic rats. Histopathological examination revealed that treatment with *P. acacia* extract, methyl gallate, and quercetin decreased degenerative changes and inflammation in the ischemic muscle. Further biochemical assessment of the hind limb tissue showed decreased oxidative stress, increased levels of nitric oxide and endothelial nitric oxide synthase (eNOS), and enhancement of the levels of heme oxygenase-1 (HO-1) and vascular endothelial growth factor (VEGF) in the groups treated with methyl gallate and quercetin. Expression levels of hypoxia inducible factor-1 alpha (HIF-1α), VEGF, fibroblast growth factor-2 (FGF-2), and miR-146a were upregulated in the muscle tissue of methyl gallate- and quercetin-treated groups along with downregulation of nuclear factor kappa B (NF-κB). In conclusion, *P. acacia* extract and its isolated compounds, methyl gallate and quercetin, mediated therapeutic angiogenesis in diabetic hind limb ischemia.

## 1. Introduction

Diabetes mellitus increases the risk of developing ischemic vascular disease, especially peripheral artery disease (PAD). PAD arises from the occlusion of blood flow in peripheral arteries, leading to ischemia and hypoxia of tissue. Neoangiogenesis, “The sprouting of new blood capillaries from preexisting vessels”, takes place in response to ischemia. However, the capability of forming new blood vessels is impaired in diabetic patients with PAD [1]. As a result, PAD in these patients may cause limb amputation and mortality [2]. Management of severe PAD is mainly through surgery and endovascular revascularization [3]. However, surgical interventions are not suitable for all diabetic patients [4]. Hence, the search for new therapeutic modalities became necessary.

Therapeutic angiogenesis is an alternate approach for improving blood flow and encouraging blood vessel growth [5]. Angiogenesis is chiefly motivated by ischemia, ischemia-induced transcription factors such as hypoxia inducible factor-1 (HIF-1), and the genes dependent on these factors, such as vascular endothelial growth factor (VEGF) [6]. Therapeutic angiogenesis was applied using cytokines to stimulate angiogenesis or transplanting cells such as endothelial progenitor cells in bone marrow to differentiate into endothelial cells and yield proangiogenic cytokines [7], but angiogenic cytokines may generate pathological vessels and cause some complications [8]. This limits the clinical use of therapeutic angiogenesis, and it is still considered a great challenge. Thus, a new strategy beyond administrating extreme amounts of angiogenic factors is needed.

Many mechanisms are involved in ischemia/reperfusion injury such as oxidative stress and mitochondrial dysfunction [9]. Heme oxygenase-1 (HO-1) is a stress-induced protective enzyme enhancing tissue regeneration. HO-1 was reported to be a mediator of VEGF-induced angiogenesis [10]. Endothelial nitric oxide synthase (eNOS) provokes many useful cardiovascular effects. Besides its vasodilatory properties, eNOS-derived nitric oxide (NO) plays a serious role in angiogenesis following tissue ischemia [11]. NO has a high affinity for heme and it strongly upregulates HO-1 [12].

The family Loranthaceae represents the largest family of the order Santalales with about 70 genera and 800 species of semi-parasitic plants commonly known as mistletoes. They tend to parasitize a broad range of gymnosperms and angiosperms causing serious damage to their hosts [13,14,15]. Despite their damaging effect on plants, they have been used in ethnomedicine for various purposes, including the treatment of diarrhea, tonsillitis, otitis media, and diabetes [16,17,18]. *Plicosepalus acacia* is a plant that belongs to the family Loranthaceae and is abundant in the Saudi kingdom. It is widely used in traditional medicine for the treatment of variable diseases and has been proved to possess anti-inflammatory, antimicrobial, hepatoprotective, and hypoglycemic functions [19,20]. A previous investigation of the chemical constituents of the plant revealed the accumulation of a number of phenolic compounds with significant antioxidant effects, of which methyl gallate and quercetin were major components [21]. The quercetin concentration was previously determined to be 0.062 g% using high-performance thin layer chromatography (HPTLC) [22]. The current study aimed to continue the previous work through HPTLC quantification of methyl gallate in addition to the identification of natural metabolites in *P. acacia* using liquid chromatography coupled to tandem mass spectrometry (LC-MS/MS). The current work also investigated the effect of the *P. acacia* extract and its isolated compounds, methyl gallate and quercetin, on therapeutic angiogenesis in experimentally induced diabetic hind limb ischemia and the possible underlying mechanisms of this effect through the assessment of ischemia-induced, antioxidant, and angiogenic markers.

## 2. Materials and Methods

### 2.1. Plant Material and Metabolic Analysis Profiling Using LC/ESI-TOF-MS/MS

#### 2.1.1. Plant Collection and Extraction

The plant was previously collected in March 2010 from Alola in the North of Saudi Arabia and was identified by Dr. Nahed Morad, Faculty of Science, King Abdulaziz University. A voucher sample was preserved and given the registration code No. 2010-PA1. The plant was air dried, finely ground followed by maceration with methyl alcohol (3 × 4000 mL) at room temperature. The total alcoholic extract was concentrated under vacuum and kept in a refrigerator.

#### 2.1.2. Metabolic Analysis Profiling Using LC/ESI-TOF-MS/MS 

High-performance liquid chromatography combined with electrospray time-of-flight tandem mass spectrometry (LC/ESI-TOF-MS/MS) was assessed as described before in detail [23,24]. In brief, 50 mg of the methanolic crude extract of *P. acacia* was dissolved in 1 mL of a solvent mixture of water: methanol: acetonitrile (50:25:25), ultra-sonicated for 10 min and then centrifuged for 10 min at 10,000 rpm. An aliquot of 50 µL was diluted with 1000 µL of the afore-mentioned solvent mixture to obtain a final concentration of 2.5 µg/µL. An amount of 10 µL was injected in both positive and negative modes. The LC-MS analysis was also repeated for blanks for confidence in the experiment. The LC/ESI-TOF-MS/MS analysis was performed on an ExionLC system (AB Sciex, Framingham, MA, USA) using an autosampler system, an in-line filter disks pre-column (0.5 µm × 3.0 mm, Phenomenex, Torrance, CA, USA), and an X select HSS T3 column (2.5 µm, 2.1 × 150 mm, Waters Corporation, Milford, MA, USA) sustained at 40 °C. The mobile phase consisted of 5 mM ammonium formate buffer in 1% methanol with pH adjusted to 3.0 using formic acid for positive mode or adjusted to 8.0 using sodium hydroxide for negative mode. The gradient elution technique was done by increasing the concentration of acetonitrile from 10% to 90% within 20 min, followed by an isocratic period of 4 min and finally reducing the concentration of acetonitrile to 10% within 3 min with a constant flow rate of 0.3 mL/min. This compartment was linked to a Triple TOF^™^ 5600+ system (AB SCIEX, Concord, NC, Canada) for MS/MS fragmentation spectra. The metabolites were identified by comparing their *m/z* and MS/MS transitions to those recorded in reference databases. Further, the retention time, molecular formula, MZmine ID, and adduct formula were detected.

### 2.2. Isolation of Methyl Gallate and Quercetin and HPTLC Analysis

#### 2.2.1. Instrumentation 

A CAMAG^®^ (CAMAG, Muttenz, Switzerland) Linomat V was utilized for sample application with adjusted conditions, slit dimension of 6 mm length and 0.1 mm width. The plates were developed in a twin-trough chamber. Each band quantification was assessed using a CAMAG TLC Scanner III densitometer and CATS version 4X software in the absorption mode using a deuterium source. 

#### 2.2.2. General Experimental Procedure

^1^H (400 MHz) and ^13^C (100 MHz) NMR spectra were recorded by a JEOL (Freising, Germany) spectrometer. Both ^1^H and ^13^C NMR chemical shifts are expressed in *δ* values regarding the solvent peaks *δ*_H_ 3.31, 4.85 and *δ*_C_ 49.1 ppm for methanol, while coupling constants are given in Hertz (Hz). The solvents (chloroform CHCl_3_, ethyl acetate EtOAc, and methanol MeOH) of HPLC grade were used for extraction, isolation, and development. The pre-coated silica gel F_254_ (0.25 mm) was purchased from Merck Company (Darmstadt, Germany) while silica gel 60/230–400 µm mesh size (Whatman^™^, Sanford, ME, USA) was used for column chromatography. Sephadex^®^ LH-20 (Sigma Aldrich, Bremen, Germany) was also utilized.

#### 2.2.3. Isolation and Purification of Methyl Gallate and Quercetin

An amount of the total alcoholic plant extract (13 g) was partitioned with EtOAc, followed by concentration under vacuum to yield 5.7 g of dry extract. The ethyl acetate extract was chromatographed by silica gel column chromatography using CHCl_3_- MeOH (100:0) up to (60:40), with step-by-step gradient elution, where three sub-fractions were obtained (Pa-EA-1 to Pa-EA-3). The spots were tracked using ferric chloride as a specific spray reagent for methyl gallate and anisaldehyde/sulfuric acid spray reagent for quercetin. The first sub-fraction (Pa-EA-1, 1.2 g) was re-chromatographed on a silica gel column with isocratic elution using CHCl_3_-MeOH (95:5), yielding two sub-fractions (Pa-EA-1-1′, Pa-EA-1-2′). The first one (0.7 g) was finally purified on a Sephadex LH-20 column eluted with CHCl_3_-MeOH (50:50) to yield 17.4 mg of white powder denoted as compound **1**. The second subfraction (Pa-EA-2, 3.7 g) was re-chromatographed on a silica gel column eluted with CHCl_3_-MeOH (85:15) to yield four sub-fractions (Pa-EA-2-1′ to Pa-EA-1-4′). The second one (1.5 g) was finally purified on a Sephadex LH-20 column eluted with MeOH to yield 31.1 mg of yellow powder denoted as compound **2**.

#### 2.2.4. HPTLC Analysis

##### Preparation of a Standard Solution of Methyl Gallate

An amount of 10 mg of the isolated methyl gallate was used to prepare a methanolic standard stock solution at a concentration of 1 mg/mL. Then, the stock solution was kept in a refrigerator to construct a calibration curve.

##### Calibration Graph

According to the requirements of the International Council on Harmonisation (ICH) guidelines [25], different concentrations of the prepared stock solution were applied to a plate with dimensions of 20 cm × 10 cm, each in triplicate with a 6-mm-band length and a distance between bands of 4 mm. The distances from both the x-axis and y-axis were 10 mm. The rate of application used was 15 μL s^−1^, and development of the bands was performed. Different solvent mixtures were used, such as chloroform-ethyl acetate (5:5), chloroform-methanol (9:1), toluene-acetonitrile (7:3), and toluene–acetonitrile (3:7). The solvent mixture consisting of toluene-acetonitrile-glacial acetic acid (3:7:0.5) was found to be the best solvent system with a suitable retention factor (R_f_). Development was performed after saturation for 20 min. The development time was 15 min, the plates were air-dried, and then R_f_ was calculated to be 0.76 ± 0.02. Quantification of the standard zones was assessed at a wavelength of λ = 254 nm, and the calibration curve was constructed. 

##### Plant Sample Assay

For determination of the methyl gallate concentration in the plant extract, 1 g of the dry extract was dissolved in 5 mL methanol, transferred to a 100 mL volumetric flask, and then the volume was adjusted with methanol. Different aliquots of the sample as well as the standard solution of the isolated methyl gallate were spotted under the above-mentioned conditions, and band areas were recorded.

### 2.3. In Vivo Study

#### 2.3.1. Animals

Male albino rats, weighing 180–220 g, were obtained from the Egyptian Organization for Biological Products and Vaccines (Vacsera, Dokki, Giza Governorate, Egypt). They were kept in plastic cages with a mesh floor and hardwood bedding under a normal light/dark cycle, with water and food provided ad libitum. Rats were left to acclimatize for one week before the experiments. 

#### 2.3.2. Induction of Diabetes

After overnight fasting, diabetes was induced by a single intraperitoneal (i.p) dose of STZ (55 mg/kg) dissolved in citrate buffer (pH 4.5) [26]. Two weeks after STZ administration, blood glucose levels were measured in samples from the tail vein using an Accu-check go blood glucose meter (Roche Diagnostic, Mannheim, Germany). Animals with blood glucose level higher than 300 mg/dL were considered diabetic and were included in the study. 

#### 2.3.3. Study Design

Forty rats were divided into five groups (8 animals each). The first group served as normal and received Tween 80. All other groups were insulted with a single (i.p.) dose of STZ (55 mg /kg). The second group was considered to be control diabetic limb ischemia. The third, fourth, and fifth groups were treated with *P. acacia* extract (300 mg/kg), methyl gallate (100 mg/kg), and quercetin (100 mg/kg), respectively. The compounds were dissolved in Tween 80. Treatment continued daily for four weeks after the onset of diabetes.

#### 2.3.4. Induction of Hind Limb Ischemia

Ischemia was induced by the tourniquet method. Rats were anaesthetized by (i.p.) injection of ketamine (75 mg/kg) and xylazine (7.5 mg/kg) [27]. The tourniquet (rubber band) was looped six times as proximally as possible on the thigh. After 4 h of ischemia, reperfusion was initiated by releasing the tourniquet [28]. After 24 h of reperfusion, animals were anaesthetized and sacrificed by cervical dislocation. 

#### 2.3.5. Collection of Tissue Samples 

The gastrocnemius muscles of all rats were removed and divided into two parts. The first part was kept at −80 °C for homogenization and the determination of different biochemical parameters. The second part was fixed in 10% formalin for histopathological investigation.

#### 2.3.6. Histopathological Investigation

Samples of the gastrocnemius muscle fixed in formalin were embedded in paraffin. From each block, sections of 3 µm thickness were submitted, mounted on a glass slide, stained with hematoxylin and eosin (H&E), and examined by an independent pathologist. Muscle tissue sections were microscopically evaluated for degenerative changes of myocytes and numbers of inter-bundles capillaries. Capillaries were counted under three different high-power fields (hpf) (200×), and the average number was calculated. The severity of microscopic lesions observed was graded based on the degree and extent of tissue damage using a four-point scale [29].

#### 2.3.7. Biochemical Analysis

##### Determination of Levels of the Oxidative Stress and Angiogenesis Markers in the Hind Limb Tissue

All oxidative stress markers were measured in the hind limb tissue homogenate of the studied groups by enzyme-linked immunosorbent assay (ELISA). Malondialdehyde (MDA) levels were measured using a Rat Malondialdehyde ELISA Kit (MyBioSource, San Diego, CA, USA, Catalog No. MBS268427). Reduced glutathione (GSH) levels were measured using a Rat Glutathione (GSH) ELISA Kit (MyBioSource, San Diego, CA, USA, Catalog No. MBS265966). Superoxide dismutase (SOD) levels were measured using a Rat Superoxide Dismutase ELISA Kit (MyBioSource, San Diego, CA, USA, Catalog No. MBS036924), and catalase levels were assessed using a Rat Catalase ELISA Kit (MyBioSource, San Diego, CA, USA, Catalog No. MBS726781), in accordance with the manufacturer’s instructions. 

Levels of NO and eNOS were assessed in the hind limb tissue lysate of the studied groups by ELISA according to the manufacturer’s instructions using a Rat Nitric Oxide (NO) ELISA Kit (MyBioSource, San Diego, CA, USA, Catalog No. MBS3808496) and a Rat Endothelial Nitric Oxide Synthase ELISA Kit (MyBioSource, San Diego, CA, USA, Catalog NO. MBS721860), respectively.

Additionally, levels of HO-1 and VEGF were determined in the hind limb tissue lysate of the studied groups by a Rat Heme Oxygenase 1 ELISA Kit (MyBioSource, San Diego, CA, USA, Catalog No. MBS764989) and a Rat Vascular Endothelial Growth Factor ELISA Kit (CUSABIO, Houston, TA, USA, Catalog No. MBS724516), respectively.

##### Determination of the Expression of miRNA-146a, NF-κb, HIF-1α, VEGF, and FGF-2 in the Hind Limb Tissue by Quantitative Real-Time PCR

Total RNA, including miRNA, was extracted from the hind limb tissue of the studied groups using a Qiagen miRNeasy Mini kit (Qiagen, Hilden, Germany, Catalog No. 217004) according to the protocol supplied by the manufacturer. RNA concentration and purity were measured spectrophotometrically using a NanoDrop 1000 spectrophotometer (NanoDrop Tech, Wilmington, DE, USA). 

MicroRNA-146a expression was measured in two steps using qRT-PCR. A TaqMan^®^ microRNA reverse transcriptase kit (Applied Biosystems, Waltham, MA, USA, Catalog No.4366596) and specific miRNA primers miRNA-146a 5× and RNU6B 5× (Applied Biosystems, Waltham, MA, USA, Catalog No. 4427975) were used for the first step, reverse transcription (RT). RNU6B was used as a housekeeping gene to normalize the miRNA-146a expression. RT reactions with a final volume 15 μL were made up of 5 μL (1–10 ng) of RNA template, 7 μL master mix, and 3 μL of primer. In a Mastercycler Gradient thermocycler (Eppendorf, Hamburg, Germany), RT was performed as follows: 16 °C for 30 min, 42 °C for 30 min, 85 °C for 5 min, and then held at 4 °C. The second step, real-time PCR, was carried out in an AB 7500HT instrument with SDS Software version 2.1.1, using TaqMan^®^ Universal Master Mix 40R (Applied Biosystems, Waltham, MA, USA, Catalog No. 4440043) and TaqMan^®^ assays miRNA-146a 20× and RNU6B 20× (Applied Biosystems, Waltham, MA, USA, Catalog No. 4427975). The PCR reactions with 20 μL final volume consisted of 4 μL complementary DNA (cDNA), 10 μL 2× TaqMan^®^ Universal Master Mix, 1 μL 20× TaqMan^®^ Gene Expression assay, and 5 μL RNase-free water. All reactions were run in duplicate using the following cycling conditions: 95 °C for 10 min, followed by 40 cycles of 95 °C for 15 sec and 60 °C for 1 min.

The expression levels of nuclear factor kappa B (NF-κb), HIF-1α, VEGF, and fibroblast growth factor-2 (FGF-2) were quantified using a GoTaq^®^ 1-Step RT-qPCR System (Promega, Madison, WI, USA, Catalog No. A6020) and glyceraldehyde 3-phosphate dehydrogenase (GAPDH) as a housekeeping gene. The sequences of the primers used and the annealing temperature for each primer are illustrated in Table 1. The reaction was carried out in a 20 μL final volume, including 4 μL RNA template, 0.4 μL GoScript™ RT mix for 1-step RT-qPCR, 1 μL of each of the forward and reverse primers, 10 μL GoTaq^®^ qPCR master mix, 0.31 μL supplemental CXR reference dye, and 3.29 μL nuclease-free water. Cycling conditions were 37 °C for 15 min for reverse transcription, 95 °C for 10 min for inactivation of the reverse transcriptase enzyme, followed by 40 cycles of 95 °C for 10 sec denaturation, annealing for 30 s (annealing temperatures are listed in Table 1), and 72 °C for 30 sec extension. All real-time PCR reactions were performed in a StepOnePlus™ Real-Time PCR thermal cycling instrument (Applied Biosystems, USA). ΔΔCt and fold change were calculated, and the results were expressed as the log 2-fold change relative to the normal group (base line), where the value of the expression in the normal group was normalized to 1, and log 2 (1) = zero.

### 2.4. Statistical Analysis

Data were processed using the Statistical Package for Social Sciences, SPSS (IBM, Armonk, NY, USA), version 21.0 software. The results were expressed as the mean ± SD. One-way analysis of variance, ANOVA, followed by Bonferroni’s post hoc test for multiple comparisons, was employed for statistical analysis. A *P* value less than 0.05 was considered statistically significant. 

## 3. Results and Discussion

### 3.1. Metabolic Analysis Profiling using LC/ESI-TOF-MS/MS

**Figure 1:**
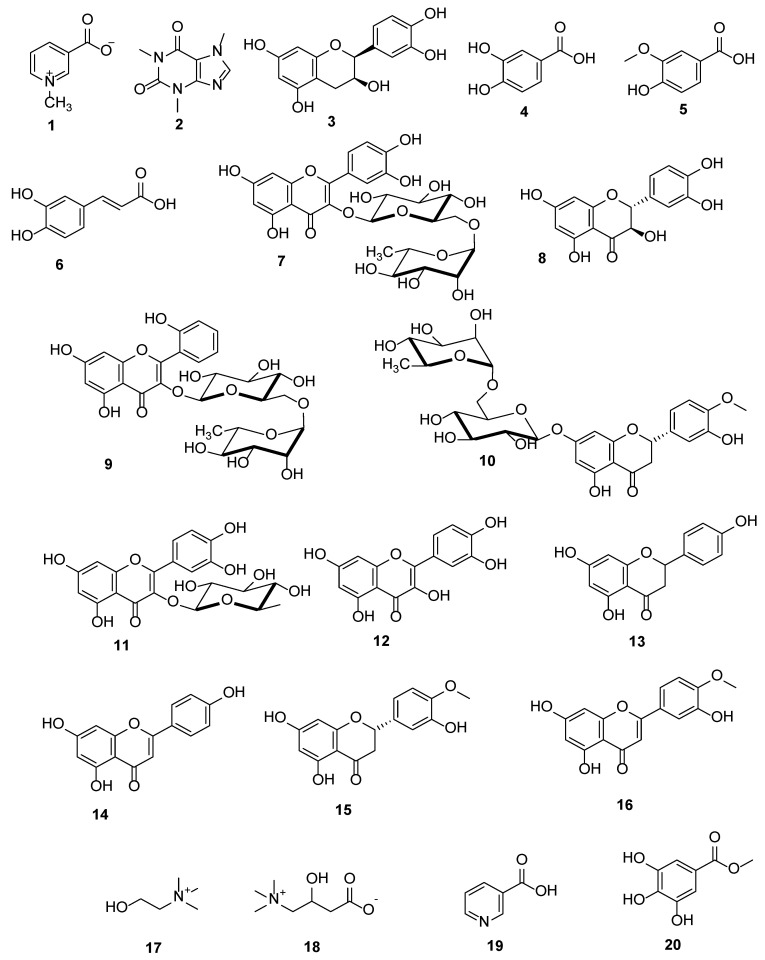
Chemical structures of the detected metabolites using LC-MS/MS, trigonelline (**1**), caffeine (**2**), epicatechin (**3**), protocatechuic acid (**4**), vanillic acid (**5**), caffeic acid (**6**), rutin (**7**), taxifolin (**8**), datiscin (**9**), hesperidin (**10**), quercetrin (**11**), quercetin (**12**), naringenin (**13**), apigenin (**14**), hesperetin (**15**), diosmetin (**16**), choline (**17**), carnitine (**18**), nicotinic acid (**19**) and methyl gallate (**20**).

Liquid chromatography combined with tandem mass spectrometry (LC-MS/MS) is a modern tool for the detection of natural metabolites that may have beneficial therapeutic effects. Herein, LC-MS/MS of the methanolic crude extract of *P. acacia* is performed for the first time (Figures S1 and S2). This metabolic analysis showed the existence of various chemical classes of natural products such as alkaloids, catechins, flavonoids, flavonoid glycosides, phenolic acids, and minor vitamins and nutrients (Table 2, Figure 1). 

Obviously, the methanolic extract of *P. acacia* is a rich source of polyphenolic compounds that are correlated with the reported antioxidant and anti-inflammatory effects of *P. acacia* [45]. The detected natural products were identified based on their mass accuracy expressed in parts per million (ppm) in addition to matching their fragmentation pattern or MS/MS ion transitions with that reported in literature [46]. Polyphenols have free radical scavenging, antioxidant, anti-aging, hepatoprotective, and reno-protective activities [47,48]. The antioxidant activity of quercetin, taxifolin, catechin, hesperetin, and naringenin was previously assessed through their capability to perform hydrogen peroxide scavenging activity, their lower metal-reducing ability, and their lipid peroxidation inhibitory activity [49]. The hypolipidemic and hypoglycemic effects of quercetin were reported through its ability to ameliorate insulin resistance, lower plasma triglyceride levels, and reduce liver fat accumulation [50]. Flavonoids acts as anti-neoplastic agents through their antioxidant activity, carcinogen scavenging, antiproliferation, apoptosis induction, inhibition of angiogenesis, and weakening of chemotherapeutic agents’ resistance [51]. On the other hand, trigonelline alkaloid and protocatechuic acid showed hypoglycemic, antimicrobial, anti-tumor, and anti-inflammatory activities [52,53]. Further, L-carnitine acts as a fat burner that induces the metabolism of lipids and decreases the serum LDL-cholesterol level without any effect on the HDL-cholesterol level [54]. Finally, the antioxidant, free radical scavenging, anti-inflammatory, and hypoglycemic therapeutic effects of the crude extract of *P. acacia* may be correlated with the metabolites detected in the current study.

### 3.2. Identification of the Isolated Compounds

**Figure 2:**
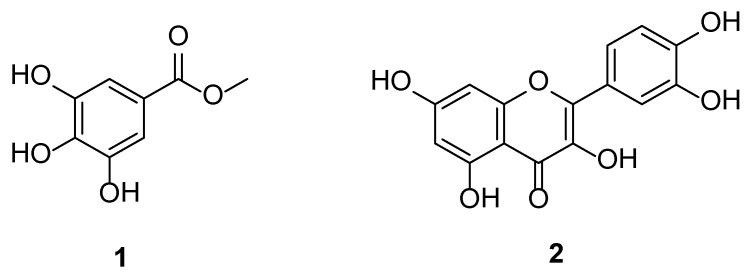
Chemical structures of the isolated methyl gallate (**1**) and quercetin (**2**).

Compound **1** (Figure 2) was obtained as a white powder, and its molecular formula was determined to be C_8_H_9_O_5_ by ESI-MS with *m/z* 185 [M + H] ^+^. The ^13^C NMR spectral data (Figure S3): δ_C_ 52.3 (OCH_3_), 110.0 (C-2,6), 121.4 (C-1), 139.7 (C-4), 146.4 (C-3,5), 168.9 (C=O). The ^1^H NMR spectral data (Figure S4): *δ*_H_ 3.80 (OCH_3_, s), 7.05 (2H, s, H-2,6). The combined spectral data together with comparison of the data previously reported in the literature [21] confirmed the structure of compound **1** as methyl gallate. 

Compound **2** (Figure 2) was obtained as a yellow powder, and its molecular formula was determined to be C_15_H_10_O_7_ by ESI-MS with *m/z* 301 [M − H]^−^. Compound **2** was identified as quercetin, which was compared with a standard.

### 3.3. HPTLC Analysis for Quantification of Methyl Gallate

#### 3.3.1. Linearity

**Figure 3:**
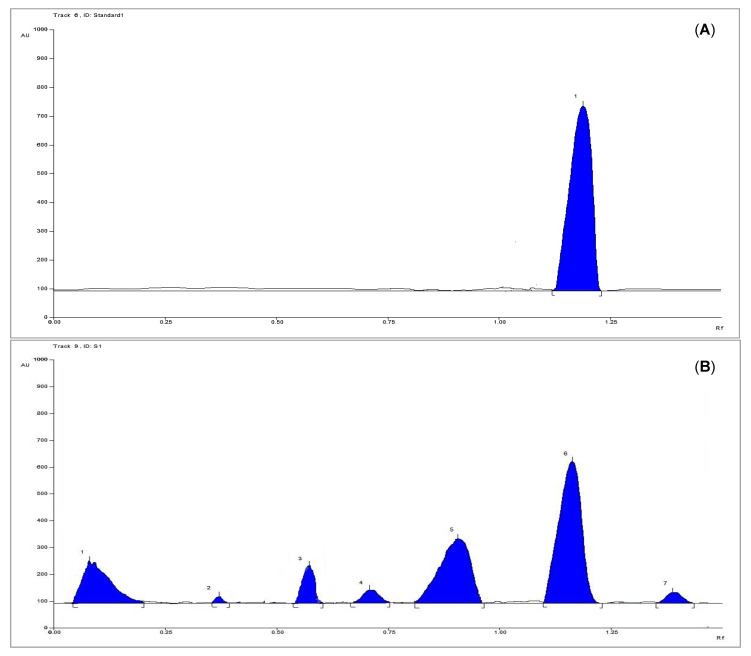
(**A**) HPTLC chromatogram of 6 µg per band of standard methyl gallate scanned at λ = 254 nm, (**B**) HPTLC chromatogram of 300 µg per band of the plant extract scanned at λ = 254 nm.

The linearity of the HPTLC method was evaluated by analyzing different six concentrations of the isolated methyl gallate as a standard solution, each in triplicate (Figure 3A). A good linear relationship was shown over the concentration range of 4–40 µg per band with respect to the area. The correlation coefficient (R^2^) was 0.966, and the linear regression equation was found to be y = 910.96x + 20,008, where y is the spot area, and x is the concentration of methyl gallate. 

#### 3.3.2. System Precision

The system precision was checked by determination of a selected concentration (2 mg/mL), applied as a 4 µg/band of the methyl gallate where it was applied in triplicate. The value of the percent relative standard deviation (%RSD) was calculated to be 2.52 and 1.44, respectively, indicating the precision (Table 3). 

#### 3.3.3. Method Precision

The plant extract was applied six times under the same above-mentioned procedure to ensure the method precision. The low value of %RSD (1.14) indicated the precision of the method (Table 3).

#### 3.3.4. Accuracy

The accuracy of the proposed method was determined by mixing of the pre-analyzed sample with a certain concentration of standard methyl gallate solution, and then, the fortified sample was investigated under the same above-mentioned procedure. The results illustrated in Table 3 indicated the accuracy of the procedure.

#### 3.3.5. Limits of Detection and Quantification

The computation of both the limit of detection and the limit of quantification were assessed according to the following equations: 3 σ/S and 10 σ/S, respectively, where σ is the standard deviation of the response and S is the slope of the calibration curve. The data are listed in Table 3. 

#### 3.3.6. Analytical Solution Stability

The analytical procedure was repeated after storage of the standard solution either at 4 °C for 10 days or at ambient temperature for two days to ensure the stability of the solutions under these conditions.

#### 3.3.7. Sample Analysis

The proposed method was applied for the determination of methyl gallate in the plant extract, which was applied as bands in triplicate (Figure 3B). The concentration of the sample was defined based on the regression equation, where the concentration was found to be 1.7 mg/100 mg of plant dry extract.

### 3.4. In Vivo Study 

#### 3.4.1. Histopathological Changes in the Study Groups

In the current study, a rat model of type 1 diabetes mellitus with hind limb ischemia was developed. This model is used widely to conduct experimental studies on PAD that is considered one of the most common complications of diabetes. It is one of the most essential models to study therapeutic angiogenesis and/or arteriogenesis [55], which is the focus of our study.

**Figure 4:**
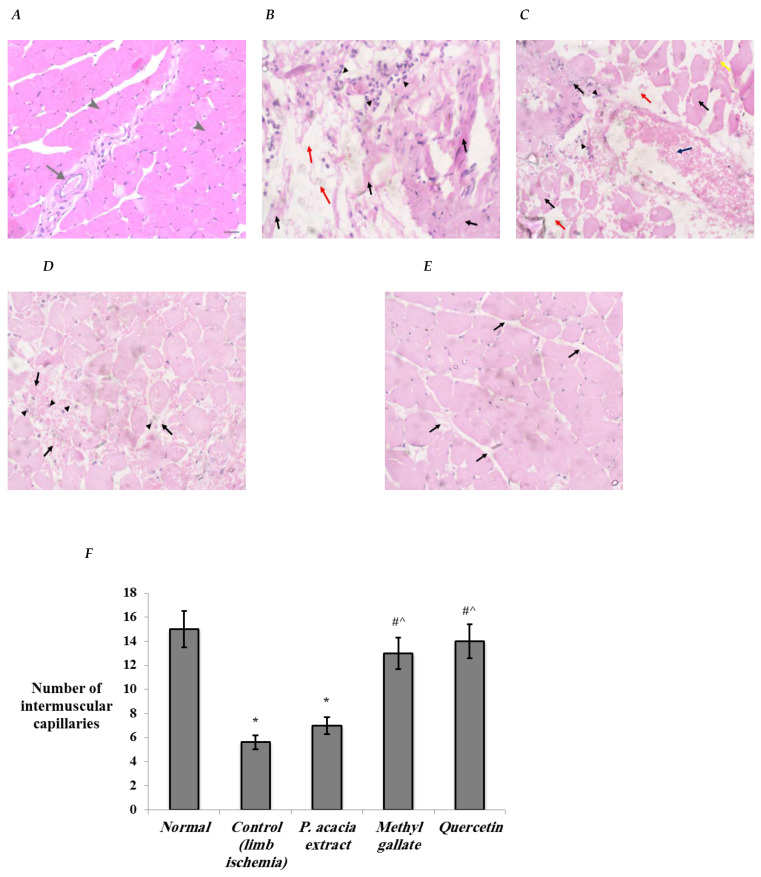
Histopathological investigation of the effects of the *P. acacia* extract and its isolated compounds (methyl gallate and quercetin) on the gastrocnemius muscle in the experimental rats. (**A**) normal group, (**B**) hind limb ischemia control group, (**C**) *P. acacia* extract-treated group, (**D**) methyl gallate-treated group, and (**E**) quercetin-treated group. (**F**) Bar charts representing the count of intermuscular capillaries. Data are expressed as the mean ± SD. Data were analyzed using one-way ANOVA followed by Bonferroni’s post hoc test. ^*^ Significantly different compared to the normal group, ^#^ significantly different compared to the hind limb ischemia control group, ^ significantly different compared to the group treated with the *P. acacia* extract. Values were considered significantly different at *p* < 0.05.

Histopathological examination of the hind limb muscle in the study groups revealed that the muscles of the normal group showed a normal bundle of skeletal muscle fibers (arrow heads) separated by thick fibrous seta containing blood vasculature (arrow) (Figure 4A). Muscle tissue of the control diabetic limb ischemia group displayed separation of bundles, degenerated necrotic muscle bundles with no identified nuclei (Black arrows), interstitial edema (Red arrows), and chronic inflammatory cells (Arrow heads) (Figure 4B). In comparison to the control group, some compact muscle bundles, degenerated necrotic muscle bundles with no identified nuclei (Black arrows), intermuscular edema (Red arrows), hemorrhage (Yellow arrow) congested vessel (Blue arrow), and chronic inflammatory cells (Arrow heads) were observed in the group treated with the *P. acacia* extract (Figure 4C). The group treated with methyl gallate showed uniform muscle bundles with mild interstitial edema and hemorrhage (Black arrows). Very few inflammatory cells (Arrow heads) were seen, but no degenerated or necrotic muscle cells (Figure 4D). The group treated with quercetin presented uniform muscle bundles with mild interstitial edema (Black arrows) (Figure 4E).

The number of intermuscular capillaries was significantly decreased in the control limb ischemia group compared to normal. Treatment with methyl gallate and quercetin displayed significant increases in numbers of intermuscular capillaries compared to the control limb ischemia and *P. acacia* extract groups (Figure 4F). The increase in the number of intermuscular capillaries/vessels indicates enhanced therapeutic angiogenesis in hind limb ischemia models [56,57].

Examined muscle tissues of the study groups were graded according to the presence of interstitial edema, hemorrhage, myocyte vacuolization, myocyte necrosis, mononuclear cell infiltration, and fibrosis (Table 4). Degenerative changes, edema, hemorrhage, and inflammation were marked in the control hind limb ischemia muscles but were moderate in the group treated with the *P. acacia* extract. Methyl gallate-treated groups exhibited mild edema, hemorrhage, and inflammation with no degenerative changes. Absence of degenerative changes and inflammation with minimal edema were detected in the group treated with quercetin (Table 4).

#### 3.4.2. Effect of Treatment with the *P. acacia* Extract, Methyl Gallate and Quercetin on the Investigated Biochemical Parameters

The hypoxia that accompanies hind limb ischemia has a strong influence on the biochemical processes in the affected tissue. It induces the generation of reactive oxygen species (ROS) [58] and leads to the development of oxidative stress, a major signal in ischemia/reperfusion injury [59]. Oxidative stress plays an essential role during angiogenesis. Physiological or pathological angiogenesis originates by tissue demands for oxygen and nutrients leading to a hypoxia/reoxygenation cycle [60]. Studies have shown that situations of hypoxia followed by reoxygenation accelerate tubular morphogenesis in human microvascular endothelial cells and encourage angiogenesis in animal models [61].

The main mechanism of oxidative stress-induced angiogenesis involves HIF/VEGF signaling [60]. ROS-mediated angiogenesis is strongly connected with VEGF expression [62]. Moreover, ROS affect VEGF-stimulated VEGFR2 dimerization and autophosphorylation, which are required for VEGFR2 activation and subsequent angiogenesis [63]. Similarly, products of oxidation are reported to interact with VEGFR2 and thus stimulate angiogenesis [64]. The augmentation of angiogenesis by oxidation products was attributed to activation of the HIF/VEGF pathway [65]. HIF-1α inhibition abrogates the proangiogenic effect of oxidized products in vivo, suggesting that the HIF-1α pathway is a possible molecular mechanism by which they induce angiogenesis [66]. 

Additionally, VEGF signaling through VEGFR2 promotes eNOS activity through multiple mechanisms. eNOS produces NO, an important vasodilator that rapidly diffuses throughout the endothelium. NO activates the enzyme soluble guanylate cyclase (sGC) to produce cyclic guanosine monophosphate (cGMP). This particular part of the eNOS signaling pathway contributes to both an acute vasodilating effect in the neighboring vascular smooth muscle cells (VSMCs) and the long-term angiogenic functions of ECs, such as proliferation [67].

Studies have also shown other mechanisms of ROS-activated angiogenesis that operate in a VEGF-independent manner. One of those mechanisms includes the formation of new lipid oxidation products with proangiogenic activities. These products function through activation of the toll-like receptor (TLR)2/MyD88 pathway, leading to the activation of Rac1 to stimulate cell migration and angiogenesis [68]. Another study reported that peroxidized unsaturated fatty acids stimulate TLR4 signaling in endothelial cells, leading to NF-κB activation [69]. Under most conditions, angiogenesis is closely related to the mobilization of inflammatory cells. During physiological or repair processes, the inflammation process is transient and acts as a substantial source of ROS [70]. In addition, TLR signaling has been shown to affect the HIF/VEGF pathway of angiogenesis. TLR2 modulates the expression of HIF-1α, which activates the VEGF promoter in response to hypoxia [71].

**Figure 5:**
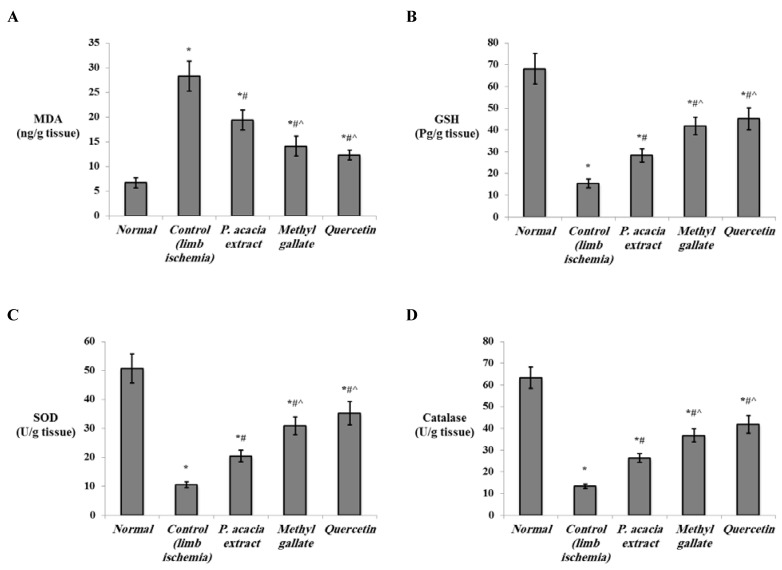
The effect of the *P. acacia* extract and its isolated compounds (methyl gallate and quercetin) on the levels of (**A**) MDA, (**B**) GSH, (**C**) SOD enzyme, and (**D**) catalase enzyme in the hind limb tissue of the experimental rats (*n* = 8). MDA = malondialdehyde; GSH = reduced glutathione; SOD = superoxide dismutase. Data are expressed as the mean ± SD and were analyzed using one-way ANOVA followed by Bonferroni’s post hoc test. * Significantly different compared to the normal group, ^#^ significantly different compared to the hind limb ischemia control group, ^ significantly different compared to the group treated with the *P. acacia* extract. Values were considered significantly different at *p* < 0.05.

In the current work, levels of MDA were elevated in the skeletal muscles of the hind limb ischemia of untreated rats, with a significant reduction of the levels of GSH, SOD, and catalase (*p* < 0.001). Treatment with the extract of *P. acacia*, methyl gallate, and quercetin significantly decreased levels of MDA and enhanced levels of GSH, SOD, and catalase in the muscle relative to the control lower limb ischemia group. The improved oxidative profile was more significantly detected in the groups that were treated with isolated quercetin and methyl gallate compared to the group treated with the *P. acacia* extract (Figure 5).

Lipid peroxidation, indicated by MDA, was reported to increase in the muscle of the limb ischemia/reperfusion model [72]. Reduced levels of GSH were also observed in hind limb ischemia-reperfusion injury in mice [73]. On the other hand, overexpression of catalase prevented the high fat diet-induced ischemic limb necrosis, myopathy, and mitochondrial dysfunction [74]. Similarly, overexpression of extracellular SOD was shown to protect against brain injury induced by chronic hypoxia [75]. 

Treatment with methyl gallate and quercetin in the present study restored the levels of the antioxidant enzymes and alleviated the oxidative stress in the hind limb ischemia muscle. The antioxidant capabilities of the studied compounds are well-established. The antioxidant properties of methyl gallate were proved in various studies [76,77,78,79]. Methyl gallate was reported to be a potent antioxidant that inhibits oxidative stress in human adipocytes [80]. The antioxidant capabilities of methyl gallate also helped to attenuate doxorubicin-induced cardiotoxicity in rats [81]. 

The antioxidant activities of quercetin have been studied extensively and have potentiated its use in several medicinal applications [82]. Quercetin therapy was reported to increase GSH levels and enhance the antioxidant capacity in rats with renal ischemia/reperfusion injury [83]. Quercetin was also reported to protect against lipopolysaccharide-induced toxicity by increasing the levels of SOD and catalase and reducing the levels of MDA [84,85].

**Figure 6:**
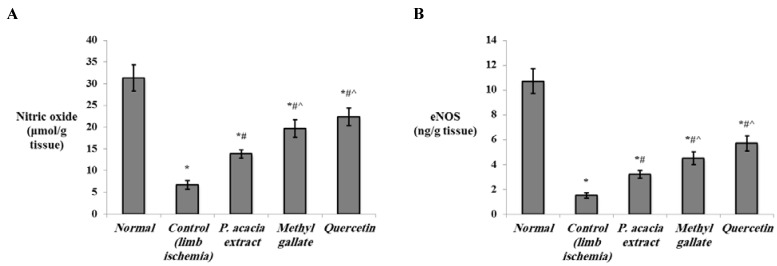
The effect of the *P. acacia* extract and its isolated compounds (methyl gallate and quercetin) on the levels of (**A**) nitric oxide, and (**B**) eNOS in the hind limb tissue of the experimental rats (*n* = 8). eNOS = endothelial nitric oxide synthase. Data are expressed as the mean ± SD and were analyzed using one-way ANOVA followed by Bonferroni’s post hoc test. * Significantly different compared to the normal group, ^#^ significantly different compared to the hind limb ischemia control group, ^ significantly different compared to the group treated with the *P. acacia* extract. Values were considered significantly different at *p* < 0.05.

Additionally, the current results revealed that the levels of NO and eNOS were significantly decreased in the hind limb ischemia muscles compared to the normal group (*p* < 0.001). Both NO and eNOS were significantly improved by treatment with *P. acacia*, methyl gallate or quercetin. Notably, levels of NO and eNOS in the hind limb ischemia muscles of the groups that received methyl gallate and quercetin were significantly higher than the levels in the group treated with *P. acacia* (Figure 6).

The expression of eNOS was previously shown to be significantly reduced in the endothelial cells of mice/rats with chronic hind limb ischemia [86,87]. The angiogenic activity of NO has been used in the regeneration of damaged tissue. Recently, pharmaceutical formulations that release NO in controlled doses were applied to enhance the process of angiogenesis [88]. In particular, a NO releasing hydrogel has been documented to enhance the therapeutic effects of mesenchymal stem cell therapy for hind limb ischemia [89]. In this context, overexpression of eNOS was reported to be protective against ischemia/reperfusion injury [90]. The eNOS-derived NO was proven to be essential for both early and late vascular recovery in response to hind limb ischemia [91]. In the current study, the enhancement of the levels of NO and eNOS was most pronounced in the group treated with quercetin. In agreement, quercetin was shown to improve endothelial function by inducing eNOS activity in human aortic endothelial cells [92]. Quercetin rapidly phosphorylates eNOS through a cAMP/protein kinase A (PKA)-mediated pathway to enhance the release of NO and to induce vasodilation [93].

**Figure 7:**
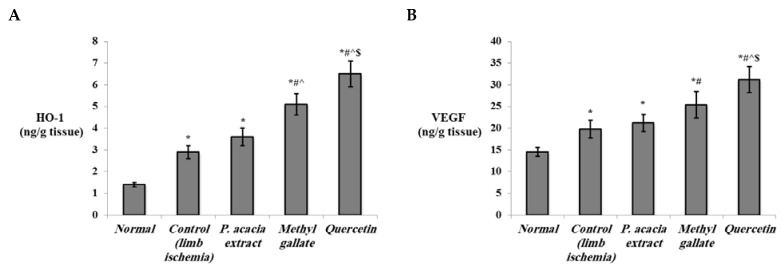
The effect of the *P. acacia* extract and its isolated compounds (methyl gallate and quercetin) on the levels of (**A**) HO-1 and (**B**) VEGF in the hind limb tissue of the experimental rats (*n* = 8). HO-1 = heme oxygenase-1; VEGF = vascular endothelial growth factor. Data are expressed as the mean ± SD and were analyzed using one-way ANOVA followed by Bonferroni’s post hoc test. * Significantly different compared to the normal group, ^#^ significantly different compared to the hind limb ischemia control group, ^ significantly different compared to the group treated with the *P. acacia* extract, ^$^ significantly different compared to the group treated with methyl gallate. Values were considered significantly different at *p* < 0.05.

**Figure 8:**
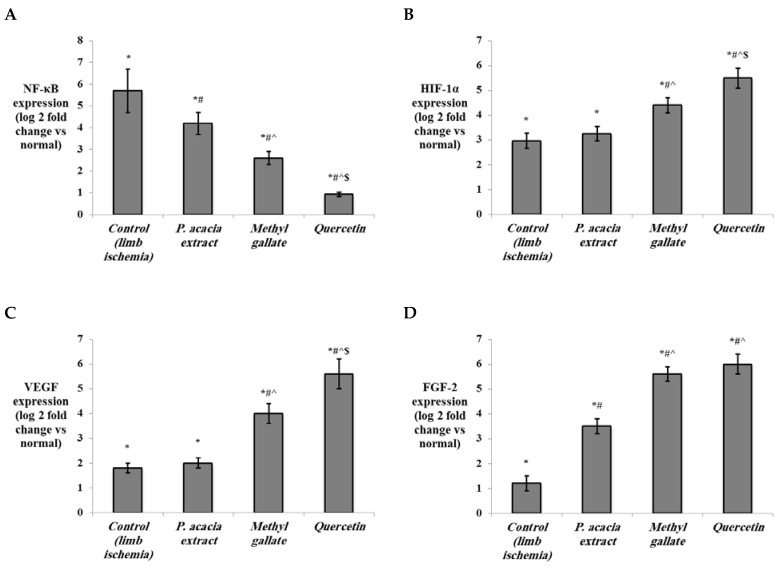
The effect of the *P. acacia* extract and its isolated compounds (methyl gallate and quercetin) on the expression levels of (**A**) NF-κB, (**B**) HIF-1α (**C**) VEGF, and (**D**) FGF-2 in the hind limb tissue of the experimental rats. (*n* = 8). NF-κB = nuclear factor kappa B; HIF-1α = hypoxia inducible factor-1 alpha; VEGF = vascular endothelial growth factor; FGF-2 = fibroblast growth factor-2. Data are expressed as mean ± SD of the log 2 fold change relative to the normal group (log 2 (1) = zero, i.e., base line). Data were analyzed using one-way ANOVA followed by Bonferroni’s post hoc test. * Significantly different compared to the normal group, ^#^ significantly different compared to the hind limb ischemia control group, ^ significantly different compared to the group treated with the *P. acacia* extract, ^$^ significantly different compared to the group treated with methyl gallate. Values were considered significantly different at *p* < 0.05.

The current study showed that the levels of HO-1 and VEGF in the ischemic muscles (Figure 7), as well as the gene expression levels of HIF-1α, VEGF, and FGF-2 (Figure 8), were significantly elevated in the control lower limb ischemia untreated rats (*p* < 0.001). The levels of all those markers were further upregulated in all treated groups, with the highest levels observed in quercetin-treated rats. In contrast, the expression of NF-κB was significantly elevated in the control lower limb ischemia group vs. the normal group (*p* < 0.001). Treatment with the extract of *P. acacia*, methyl gallate, and quercetin significantly downregulated the expression of NF-κB in the lower limb ischemic muscles compared to the control group. This downregulation was most obvious in the quercetin-treated group, where the expression levels of NF-κB in were significantly lower than in the two other treated groups (Figure 8).

HO-1 is a rate-limiting enzyme that catalyzes oxidative degradation of heme to iron, biliverdin, and carbon monoxide. It is induced under stressful conditions, involving hypoxia [94]. HO-1 is a downstream target of the hypoxia-inducible transcription factor HIF-1α, the key regulator of the body’s response to hypoxia [95]. It was recently reported that skeletal muscle HO-1 stabilizes HIF-1α through its carbon monoxide product and promotes effective glucose utilization to protect against ischemia-mediated tissue injury and necrosis [96]. The protective role of HO-1 against other types of ischemia such as renal [97] and myocardial ischemia [98] by reducing oxidative stress has also been documented. Moreover, HO-1 has been identified as an important player in cellular defense against stressful conditions. HO-1 protects endothelial cells from apoptosis, regulates vascular tone, relaxes blood vessels, attenuates inflammatory responses in the vessel wall, and participates in blood vessel formation via angiogenesis and vasculogenesis [99].

Upregulation of the pro-angiogenic factors (VEGF and FGF-2) as well as HIF-1α and HIF-2α promotes angiogenic, glycolytic, erythropoietic, and anti-apoptotic pathways in the treatment of several ischemic disorders [100]. HIFs induce a wide range of adaptive responses, the majority of which focus on upregulation of transcriptional cascades that are important for tissue protection and adaptation [101]. This includes the upregulation of glycolytic genes such as phosphoglycerate kinase (PGK) and lactate dehydrogenase A (LDHA), both of which help the tissues to adapt to low oxygen levels and anerobic ATP generation. Further, HIFs stimulate erythrocyte proliferation via erythropoietin (EPO) and angiogenesis via vascular endothelial growth factor (VEGF), which are important to improve oxygen supply to the hypoxic region. Of the two isoforms of HIFs, HIF-1 regulates the majority of these processes and is expressed systemically [100].

The role of HIF-1α stabilization in the management of ischemic diseases is vital. Adenoviral transfer of HIF-1α to a model of critical limb ischemia secondary to diabetes decreased tissue necrosis, activated eNOS, and enhanced the recovery of limb perfusion and function [102]. The HIF-1 pathway is a major regulator of hypoxia-induced angiogenesis, in which case it acts in synergy with VEGF, the master regulator of angiogenesis [103]. VEGF is a major transcriptional target for HIF-1 [104], where VEGF was found essential for the HIF-1 mediated neovascularization [105]. In ischemic stroke, VEGF was found to possess potent anti-inflammatory effects, promote angiogenesis, and protect ischemic neurons from injury [106]. VEGF mediates proliferation, migration, and survival of endothelial cells through upregulation of mitogen-activated protein kinase (MAPK) pathways [107]. A clinical trial that involved the delivery of VEGF on a plasmid resulted in effective collateral formation of blood vessels in ischemic limbs [108]. 

Another promising pro-angiogenic factor is FGF-2 that contributes to angiogenesis in synergy with VEGF by inducing proliferation and migration of endothelial cells [107]. FGF-2 is upregulated in response to hypoxia where it interacts with HIF-1α [109]. Importantly, NO stabilizes HIF-1α and stimulates secretion of VEGF and FGF-2 from endothelial cells and macrophages, leading to induction of angiogenesis [110]. In experimental critical limb ischemia, therapeutic angiogenesis was mediated by combined actions of VEGF and FGF-2 [111]. FGF-2 was reported to inhibit endoplasmic reticulum stress and attenuate renal ischemia reperfusion injury [112].

In line with the current findings, quercetin was reported to promote angiogenesis in ischemic tissue but not in tumor tissues [113]. This might be explained by its ability to upregulate HO-1 [114]. Quercetin has been listed as a natural inducer of HO-1 via the MAPK/the nuclear factor erythroid 2–related factor 2 (Nrf2) pathways [115,116]. Nrf2 negatively controls the NF-κB signaling pathway. Nrf2 induces increases in the cellular expression of HO-1, leading to inhibition of the oxidative stress-mediated NF-κB activation. Additionally, Nrf2 negatively regulates the inhibitor kappa B-alpha (IκB-α) proteasomal degradation and nuclear translocation of NF-κB [117]. This matches with our results, where the expression of NF-κB was significantly downregulated in the treated groups.

NF-κB was found to be a transcriptional activator of HIF-1α, linking ischemia to innate immunity and inflammation [104]. Inhibition of TLR4 and NF-κB pathways was involved in amelioration of acute kidney injury induced by limb ischemia/reperfusion [118]. Similar results were reported in lung injury induced by limb ischemia where suppression of TLR-4 and NF-κB had a protective effect [119]. Quercetin is known to possess anti-inflammatory properties by inhibiting the transcriptional activity of NF-κB [120].

**Figure 9:**
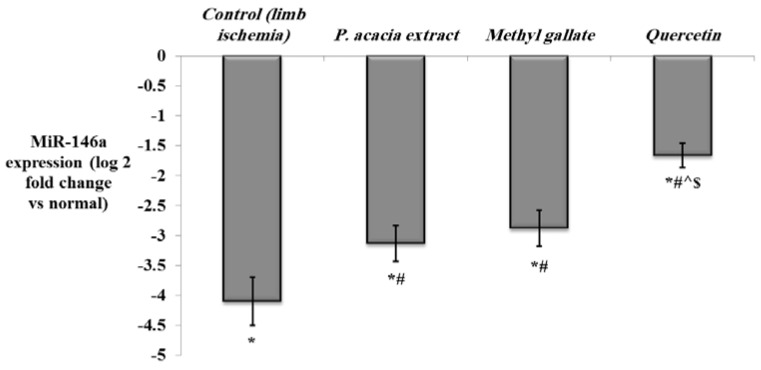
The effect of the *P. acacia* extract and its isolated compounds (methyl gallate and quercetin) on the expression levels of miR-146a in the hind limb tissue of the experimental rats. (*n* = 8). Data are expressed as mean ± SD of the log 2 fold change relative to the normal group (log 2 (1) = zero, i.e., base line). Data were analyzed using one-way ANOVA followed by Bonferroni’s post hoc test. * Significantly different compared to the normal group, ^#^ significantly different compared to the hind limb ischemia control group, ^ significantly different compared to the group treated with the *P. acacia* extract, ^$^ significantly different compared to the group treated with methyl gallate. Values were considered significantly different at *p* < 0.05.

The results of the present study also demonstrated downregulation of miR-146a in the hind limb ischemia control rats vs. the normal group (*p* < 0.001), with significant upregulation in all treated groups, particularly quercetin (Figure 9).

The expression of miR-146a was reported to be downregulated in acute ischemic stroke [121]. Similarly, the expression of miR-146a was decreased in mice Kupffer cells following hepatic ischemia/reperfusion [122]. The upregulation of miR-146a was suggested to have a protective role against myocardial ischemia reperfusion injury [123]. Moreover, miR-146a was proved to ameliorate liver ischemia/reperfusion injury by suppressing interleukin-1 receptor-associated kinase 1 (IRAK1) and tumor necrosis factor receptor-associated factor 6 (TRAF6), leading to the inhibition of NF-κB [122]. Additionally, miR-146a expression was downregulated in small intestine ischemia. However, upregulation of miR-146a was found to have a protective role against intestinal ischemia/reperfusion injury through inhibition of the TLR4/TRAF6/NF-κB pathway [124]. Upregulation of miR-146a during angiogenesis was frequently reported. Overexpression of miR-146a in human umbilical vein endothelial cells resulted in up-regulation of angiogenesis-associated genes including FGF2 [125]. miR-146a was also reported to enhance angiogenesis of endothelial cells in hepatocellular carcinoma by promoting the expression of platelet-derived growth factor receptor α (PDGFRA) [126]. Interestingly, the levels of miR-146a were negatively correlated with inflammatory and oxidative stress markers, but positively correlated with the levels of Nrf2, HO-1, and SOD in the brain of chronic type 2 diabetic rats [127]. In agreement with our findings, quercetin was recently reported to upregulate miR-146a in fibroblast-like synoviocytes of rheumatoid arthritis [128].

In conclusion, the current study suggests a beneficial role of methyl gallate and quercetin isolated from *P. acacia* in the alleviation of lower limb ischemia induced in diabetic rats, through their antioxidant effects, along with their ability to induce angiogenesis via induction of HO-1, HIF-1α, VEGF, FGF-2, and miR-146a. This study adds novel insights into the biological activities of the studied compounds, especially methyl gallate that, despite being known as a potent antioxidant, is not yet extensively studied with respect to its biochemical and molecular effects.

## Figures and Tables

**Table 1:** Primer sequences and annealing temperatures for the measured genes.

Gene	Forward Primer	Reverse Primer	Annealing Temperature
NF-κb	5’-CAATGGCTACACAGGACCA-3’	5′-CACTGTCACCTGGAACCAGA-3′	55 °C
HIF-1α	5’-TGCTTGGTGCTGATTTGTGA-3’	5’-GGTCAGATGATCAGAGTCCA-3’	54 °C
VEGF	5’-AAAAACGAAAGCGCAAGAAA-3’	5’-TTTCTCCGCTCTGAACAAGG-3’	51 °C
FGF-2	5’-GGCTCTACTGCAAGAACGGC-3’	5’-GAAACAGTATGGCCTTCTGTC-3’	53 °C
GAPDH	5′-ATGACTCTACCCACGGCAAG−3’	5′-GATCTCGCTCCTGGAAGATG-3’	55 °C

**Table 2:** LC-MS/MS metabolic analysis of the methanolic crude extract of *Plicosepalus acacia*.

No.	Polarity Mode	MZmine ID	Ret. Time (min)	Measured *m/z*	Calculated *m/z*	Mass Error (ppm)	Adduct	Molecular Formula	MS/MS Spectrum	Deduced Compound	Ref.
Alkaloids											
1	Positive	83	1.37	138.0543	138.0555	−8.69	[M + H] ^+^	C_7_H_7_NO_2_	138, 94	Trigonelline	[30]
2	Positive	167	4.94	195.0876	195.0882	−3.08	[M + H] ^+^	C_8_H_10_N_4_O_2_	195, 138	Caffeine	[31]
Catechins											
3	Negative	272	4.63	289.0717	289.0712	1.73	[M − H] ^−^	C_15_H_14_O_6_	289, 245, 205, 179	(−)−Epicatechin	[32]
Phenolic Acids											
4	Negative	108	1.21	153.0192	153.0188	2.61	[M − H] ^−^	C_7_H_6_O_4_	153, 109	Protocatechuic acid	[33]
5	Negative	326	6.86	167.0343	167.0344	−0.60	[M − H] ^−^	C_9_H_8_O_2_	167, 152, 124, 108	Vanillic acid	[34]
6	Positive	657	9.36	181.0516	181.0501	8.82	[M + H] ^+^	C_9_H_8_O_4_	181, 163	Caffeic acid	[35]
Flavonoids and Their Glycosides											
7	Negative	307	6.26	609.1453	609.1456	−0.49	[M − H] ^−^	C_27_H_30_O_16_	609, 300	Rutin	[35]
8	Negative	314	6.50	303.0504	303.0505	−0.33	[M − H] ^−^	C_15_H_12_O_7_	303, 285	Taxifolin	[36]
9	Negative	318	6.57	593.1569	593.1506	10.62	[M − H] ^−^	C_27_H_29_O_15_	593, 285	Datiscin	[37]
10	Positive	305	6.71	611.1911	611.1976	−10.63	[M + H] ^+^	C_28_H_34_O_15_	611, 303	Hesperidin	[38]
11	Negative	339	7.22	447.0945	447.0927	4.03	[M − H] ^−^	C_21_H_20_O_11_	447, 300	Quercetrin	[37]
12	Negative	488	9.51	301.0359	301.0348	3.65	[M − H] ^−^	C_15_H_9_O_7_	301, 151	Quercetin	[35]
13	Negative	528	10.03	271.0607	271.0606	0.37	[M − H] ^−^	C_15_H_12_O_5_	271, 177, 151, 119	Naringenin	[39]
14	Negative	558	10.48	269.0465	269.0450	5.58	[M − H] ^−^	C_15_H_10_O_5_	269, 151	Apigenin	[35]
15	Positive	863	10.62	303.0886	303.0869	5.61	[M + H] ^+^	C_16_H_14_O_6_	303, 151	Hesperetin	[40]
16	Positive	975	11.42	301.0736	301.0712	7.97	[M + H] ^+^	C_16_H_12_O_6_	301, 286	Diosmetin	[41]
Miscellaneous compounds											
17	Positive	33	1.19	104.1066	104.1070	−3.84	[M] ^+^	C_5_H_14_NO	104, 60	Choline	[42]
18	Positive	37	1.24	162.1115	162.1130	−9.25	[M + H] ^+^	C_7_H_15_NO_3_	162, 103, 85	Carnitine	[43]
19	Positive	141	1.73	124.0386	124.0399	−10.48	[M + H] ^+^	C_6_H_5_NO_2_	124, 80	Nicotinic acid	[30]
20	Negative	281	4.90	183.0309	183.0293	8.74	[M − H] ^−^	C_8_H_8_O_5_	183, 140, 124	Methyl gallate	[44]

**Table 3:** Validation parameters of the HPTLC method for the determination of methyl gallate scanned at λ = 254 nm.

Parameter	Results
Linearity range (µg per band)	4–40
Correlation coefficient (R^2^)	0.966
Regression equation	Y = 910.69X + 20,008
Limit of detection (µg per band)	0.4893
Limit of quantification (µg per band)	1.6310
System precision (%RSD)	2.52
Method precision (%RSD)	1.14

RSD: relative standard deviation.

**Table 4:** Histopathological features of the examined muscle tissues.

Histopathological Features	Normal	Control (Limb Ischemia)	*P. acacia*	Methyl Gallate	Quercetin
Degenerative changes	None	Marked (Grade 4)	Moderate (Grade 3)	None	None
Edema, hemorrhage	None	Marked (Grade 4)	Moderate (Grade 3)	Mild hemorrhage (Grade 2)	Minimal edema (Grade 1)
Inflammation	None	Marked (Grade 4)	Moderate (Grade 3)	Mild (Grade 2)	None

## Data Availability

Data is available within the article and Supplementary Materials.

## Supplementary Materials

The following are available online at https://www.mdpi.com/article/10.3390/antiox10111701/s1, Figures S1 and S2: LC-ESI-HR-MS of the crude extract (positive and negative modes), Figures S3 and S4: ^1^H NMR, ^13^C NMR spectra of compound **1**.
