# Supplementary material for: Plicosepalus acacia Extract and Its Major Constituents, Methyl Gallate and Quercetin, Potentiate Therapeutic Angiogenesis in Diabetic Hind Limb Ischemia: HPTLC Quantification and LC-MS/MS Metabolic Profiling
Source: Antioxidants (Basel). 2021 Oct 27;10(11):1701. doi: 10.3390/antiox10111701 (PMC8614836; doi:10.3390/antiox10111701)
Supplement: Supplementary file 1 [file antioxidants-10-01701-s001.zip › antioxidants-1425051-supplementary.pdf]

Supplementary Materials

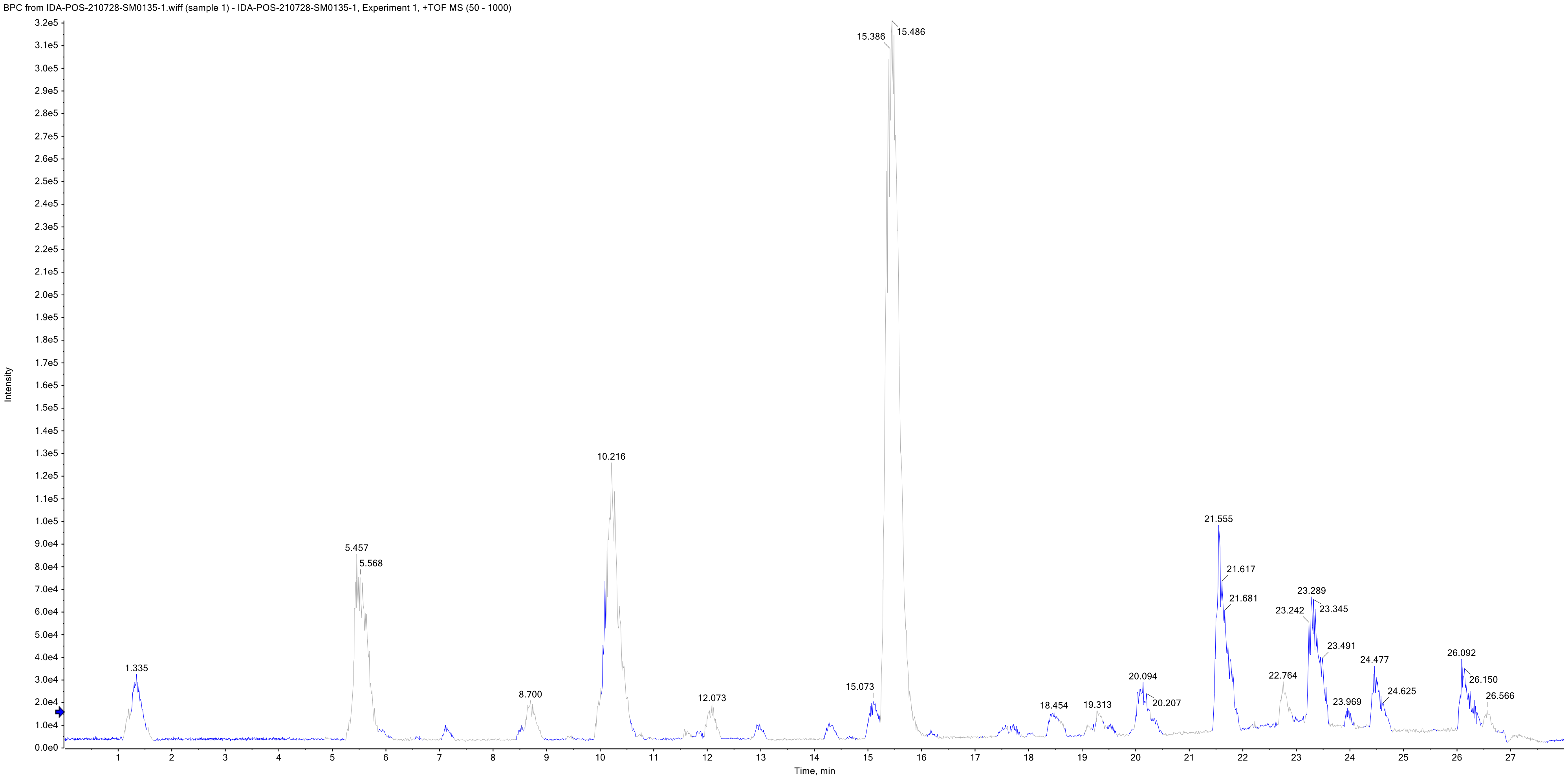

Figure S1. Chromatogram of LC-HR-ESI-MS analysis of crude extract of *Plicosepalus acacia* (positive mode).

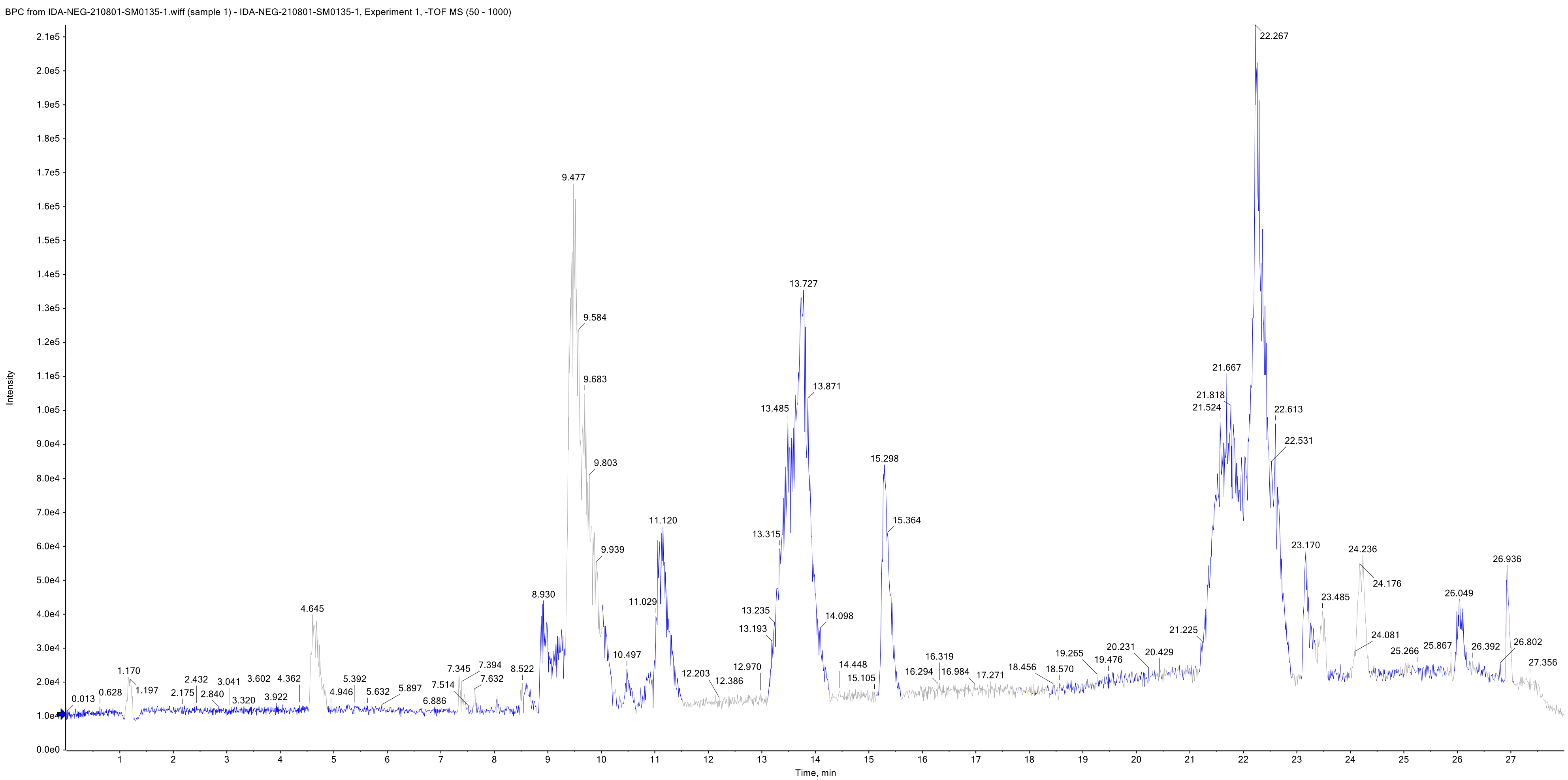

Figure S2. Chromatogram of LC-HR-ESI-MS analysis of crude extract of *Plicosepalus acacia* (negative mode).

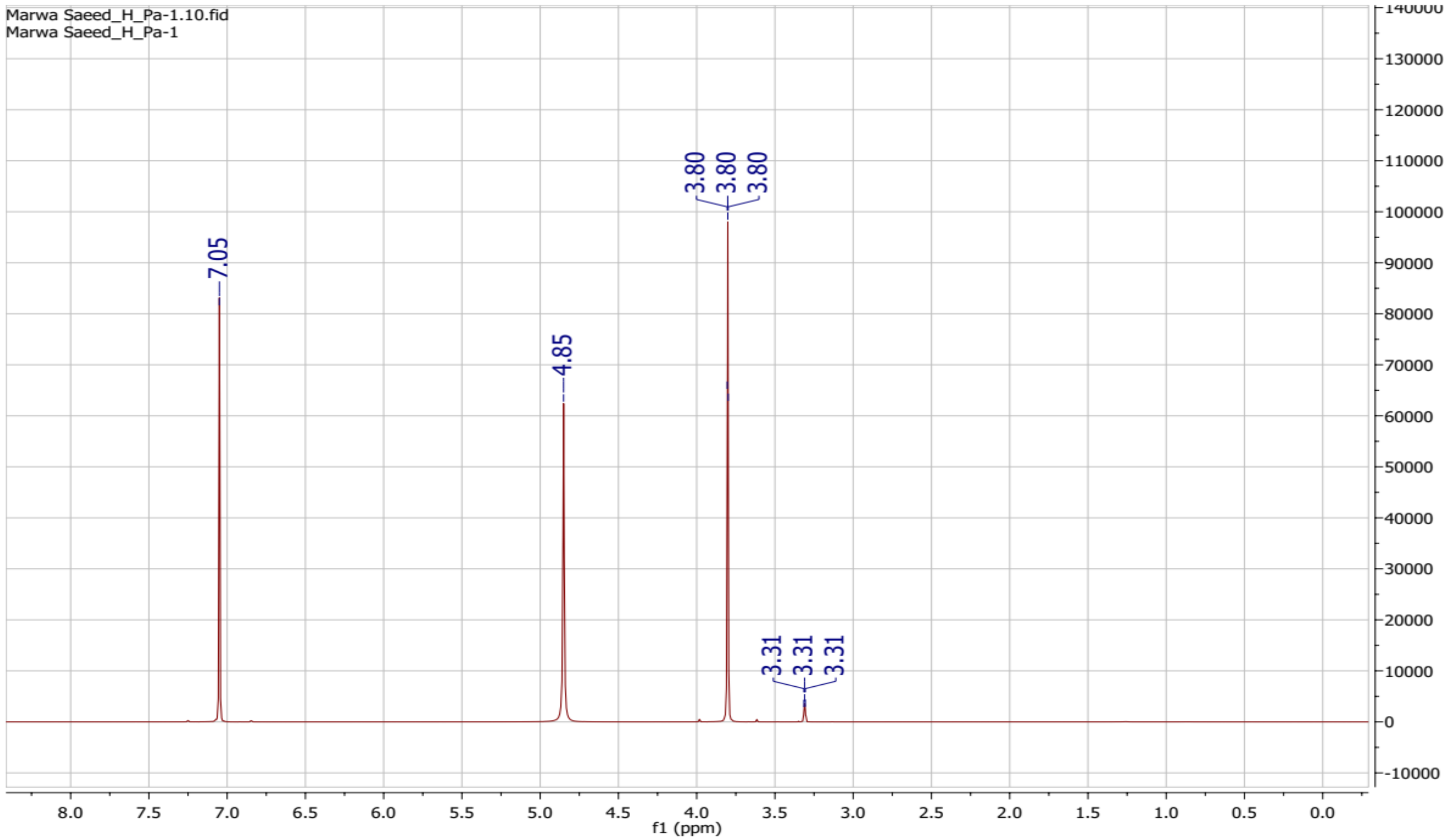

Figure S3. ^{1}H NMR spectrum of compound 1.

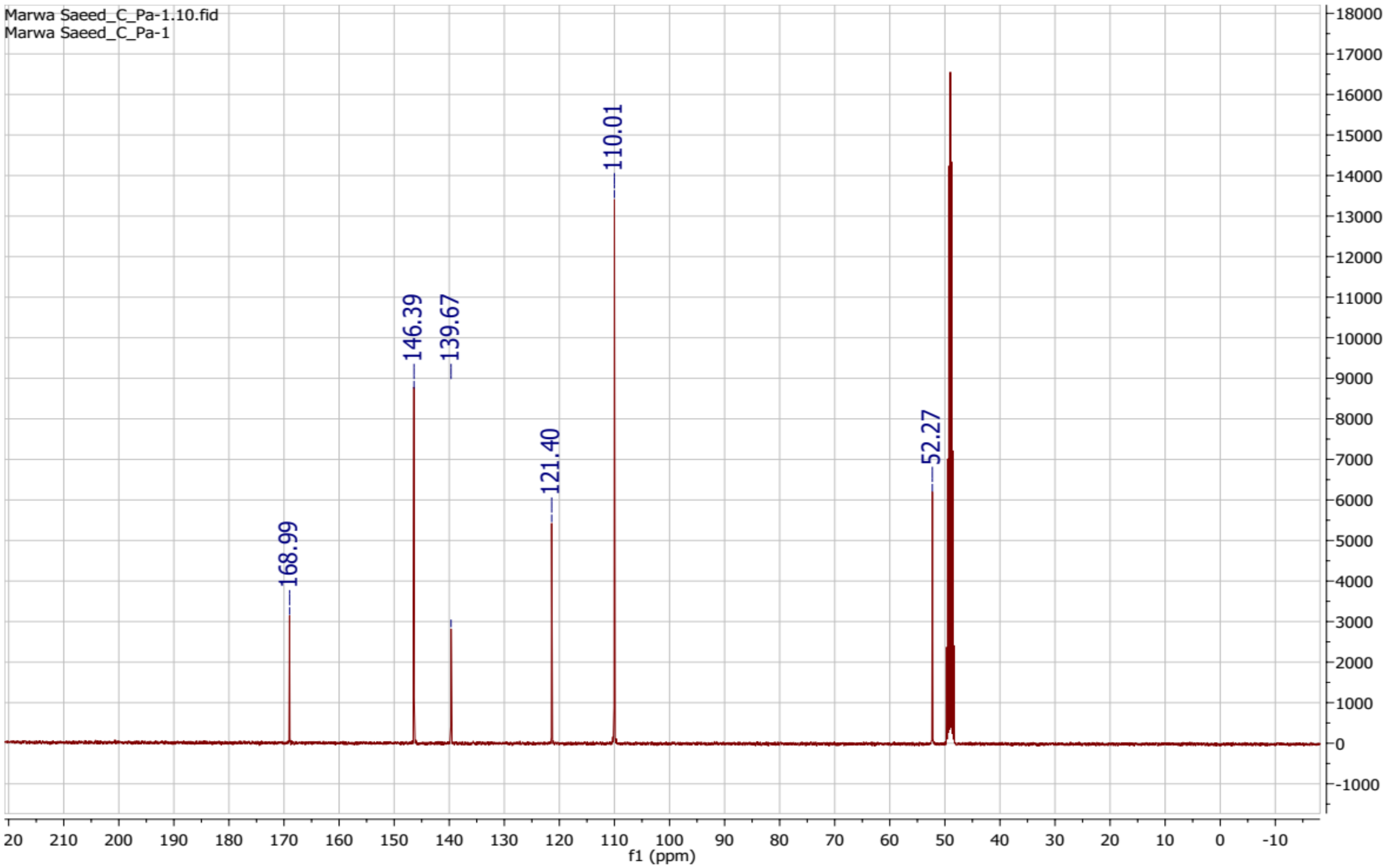

Figure S4. ^{13}C NMR spectrum of compound 1.
